# Supplementary material for: Cell kinetics during regeneration in the sponge Halisarca caerulea: how local is the response to tissue damage?
Source: PeerJ. 2015 Mar 10;3:e820. doi: 10.7717/peerj.820 (PMC4358696; doi:10.7717/peerj.820)
Supplement: Supplemental Information 3 [file peerj-03-820-s003.docx]

library(lme4)

library(lmerTest)

library(lsmeans)

#MIXEL LINEAR MODEL CHOANOCYTE PROLIFERATION IN DIFFERENT AREAS AND OVER TIME

brdu <- read.csv(file="lateregeneration.csv",head=TRUE,sep=";")

noSS = brdu[!(brdu$area=='ss'),]

time1 <- as.factor(noSS$hours)

m1 <- lmer(choan ~ area + time1 + (1 | sponge), data=noSS)

anova(m1, ddf='Kenward-Roger')

#time and area are significant

#is choanocyte proliferation over time significant between all areas?

lsmeans(m1, pairwise ~ area, adjust = 'tukey')

#LINEAR MODELS CHOANOCYTE PROLIFERATION OVER TIME IN EDGE, 1CM, 3CM

#EDGE

onlyE = brdu[brdu$area=='E',]

time2 <- as.factor(onlyE$hours)

m2 <- lm(choan ~ time2, data=onlyE)

anova(m2)

#1CM

only1cm = brdu[brdu$area=='1cm',]

time3 <- as.factor(only1cm$hours)

m3 <- lm(choan ~ time3, data=only1cm)

anova(m3)

#3CM

only3cm = brdu[brdu$area=='3cm',]

time4 <- as.factor(only3cm$hours)

m4 <- lm(choan ~ time4, data=only3cm)

anova(m4)

#CHOANOCYTE PROLIFERATION COMPARED TO STEADY-STATE SPONGES AT 6 DAYS

only6d = brdu[brdu$hours=='144',]

onlyss = brdu[brdu$hours=='0',]

ss6d <- rbind(onlyss, only6d)

m5 <- lm(choan ~ area, data=ss6d)

anova(m5)

lsmeans(m5, pairwise ~ area, adjust = 'tukey')

#MESOHYL CELL PROLIFERATION OVER TIME AND AREA

time5 <- as.factor(noSS$hours)

m6 <- lmer(mes ~ area + time5 + (1 | sponge), data=noSS)

anova(m6, ddf='Kenward-Roger')

#MESOHYL CELL PROLIFERATION COMPARED TO STEADY-STATE

m7 <- lm(mes ~ area, data=brdu)

anova(m7)
